# Effects of Cognitive, Simulator, and Real-World Training on Novice Driver Gaze Behaviour: A Pre–Post Study

**DOI:** 10.3390/jemr19030045

**Published:** 2026-04-30

**Authors:** Prem Sudhakar Lawrence, Aiswaryah Radhakrishnan

**Affiliations:** Department of Optometry, Faculty of Medical and Health Science, SRM Medical College Hospital and Research Centre, SRM Institute of Science and Technology, Chengalpattu 603203, Tamil Nadu, India; premsudl@srmist.edu.in

**Keywords:** novice drivers, eye-tracking, visual search, driving simulator, visual attention, hazard perception

## Abstract

Novice drivers demonstrate inefficient visual scanning and elevated crash risk relative to experienced drivers. Different training programmes may influence gaze behaviour and performance in distinct ways. This study compared the impact of cognitive, simulator-based, and real-world training on visual attention and driving-related outcomes in novice drivers. Thirty novice drivers (18–27 years; ≤1 year driving experience) were randomized into three training groups (*n* = 10 each): cognitive training (PsyToolkit, Version 3.7.0), game-based simulator training, and supervised real-world driving. Baseline and post-training assessments included visuomotor performance (Fitts’ Law), attentional cueing (valid/invalid reaction time), simulator-based driving errors, and eye-tracking measures of gaze behaviour. Eye-tracking outcomes included dwell-time percentage and first-fixation order across predefined areas of interest (AOIs). Participants completed 10 consecutive days of modality-specific training. Cognitive training improved visuomotor performance and increased forward road monitoring. Game-based simulator training yielded the largest reductions in simulator driving errors, particularly lane deviations (Z = −2.89, *p* = 0.004). Real-world driving altered visual scanning patterns, with significant differences in rear-view mirror prioritization (*p* = 0.024). Across groups, gaze shifted from dashboard view toward safety-relevant AOIs. Training modifies novice drivers’ gaze behaviour in modality-specific ways, suggesting that a multimodal training approach may enhance visual attention and driving safety

## 1. Introduction

Road safety is a significant public-health concern worldwide, as novice drivers exhibit a higher risk of crashes compared to experienced drivers [1,2]. This increased risk is not only due to poor vehicle-handling skills but also poor visual search strategies, poor hazard anticipation, and poor attention allocation in busy traffic situations [3,4,5]. Earlier research indicates that novice drivers often possess reduced vehicle-control skills and attentional capacity compared with experienced drivers [6,7].

Visual attention is essential for safe driving, requiring continuous monitoring of dynamic road elements such as vehicles, pedestrians, and traffic signals [8,9]. Eye-tracking methods are increasingly used to understand how drivers distribute visual attention within the driving environment. Measures such as dwell time and fixation location indicate how drivers prioritize visual information and detect relevant cues [4,10]. These measures are typically employed to distinguish between novice and experienced drivers and have been linked to hazard perception and situation awareness [5,11,12].

The present study distinguishes between three closely related constructs. Visual attention refers to the allocation of cognitive resources, gaze behaviour to observable eye movements (e.g., fixation duration and location), and visual search strategy to the temporal organization of gaze (e.g., scan order). Hazard perception is a key cognitive skill underlying crash risk and is associated with earlier fixations on hazards, anticipatory glances, and broader scanning patterns [4,12].

Recent research has explored how different training modalities—cognitive (e.g., hazard anticipation tasks), game-based simulator practice (e.g., racing video games), and real-world driving—affect the development of gaze behaviour in novice drivers [13,14]. Nevertheless, these modalities have typically been studied in isolation, and direct comparative evidence remains limited.

Different training methods can influence gaze allocation in different ways: the game-based training method has been shown to increase attention toward lane position and speed monitoring, along with faster task performance [15]. PC-based training using crash videos and structured feedback has been shown to improve hazard prediction scores, and even reduce real-world risky behaviours such as heavy braking and speeding [16], and cognitively demanding tasks can increase gaze toward the road centre and reduce peripheral scanning [17,18]. These patterns suggest that different modalities of training may influence distinct improvements in gaze allocation. However, direct comparisons across training modalities remain scarce [19].

Previous work shows that novice drivers’ gaze behaviour can be modified by different training modalities. Understanding modality-specific effects is important, as driving performance depends on perceptual, visuomotor, and hazard-monitoring processes [10,11]. Identifying modality-specific effects on gaze behaviour may assist in the development of more effective training programmes for novice drivers. Few studies have directly compared these training modalities within a unified framework using both spatial (AOI dwell time) and temporal (scan-order) eye-tracking measures.

This study aimed to compare the effects of three training modalities—cognitive task-based training, game-based simulator training, and real-world driving—on gaze behaviour and performance in novice drivers using a pre–post experimental design. Visual attention was assessed through eye-tracking measures of dwell time percentage and first fixation, while performance outcomes were quantified using Fitts’ law metrics and valid/invalid cueing tasks.

Hypothesis: It was hypothesized that cognitive training would improve visuomotor performance and increase forward gaze, simulator training would reduce driving errors and promote distributed gaze, and real-world training would enhance hazard-monitoring behaviour, reflected in increased mirror and peripheral attention.

## 2. Materials and Methods

### 2.1. Participants

The present study involved 30 novice drivers, aged between 18 and 27 years, each with one year or less of driving experience. All participants underwent a comprehensive ocular examination and were confirmed to have normal or corrected-to-normal vision. Individuals with ocular pathologies, systemic illnesses, or neurological conditions that could affect visual or cognitive performance were excluded. Participants were recruited from among students, staff, and drivers around Chennai, Tamil Nadu. The participants were randomly assigned to groups using a computer-generated simple randomization sequence and divided into 3 major categories: (1) Visual function training group, (2) Game-based training group, and (3) Real-driving training group.

The study adhered to the principles of the Declaration of Helsinki and was conducted under the approval of the Institutional Ethics Committee, SRM Medical College Hospital and Research Centre (approval No: 3080/IEC/2022). Written informed consent was obtained from all participants prior to inclusion.

Sample size was determined based on previous experimental eye-tracking studies in novice drivers, which typically include 24–36 participants. Given the within-subject pre–post design, which enhances statistical power by reducing inter-individual variability, and the inclusion of repeated testing over a 10-day training period, a total sample of 30 participants was considered sufficient to detect moderate training-related effects. Although a formal *a priori* power analysis was not conducted, this sample size was deemed adequate for detecting moderate effect sizes in the primary outcomes, consistent with prior eye-tracking research.

### 2.2. Experimental Set Up

This prospective, experimental study was designed to measure and compare the effects of training on key visual and cognitive functions of novice drivers relevant to driving performance, namely hand–eye coordination (Fitt’s Law), attentional cueing (valid and invalid), driving error measurements, and also scan order, areas of gaze distribution, and dwell time percentage before and after training.

#### 2.2.1. Visual Function Training Group Facilitated by PsyToolkit

Cognitive training tasks were implemented using the PsyToolkit platform, a specialized web-based platform designed to expedite psychological research. This platform enabled the precise assessment of cognitive processes, whose validity had been substantiated in a previous study [11]. All tasks, including the Spatial Cueing and Fitts’ law (hand–eye coordination), were configured on the PsyToolkit online platform.

**Figure 1:**
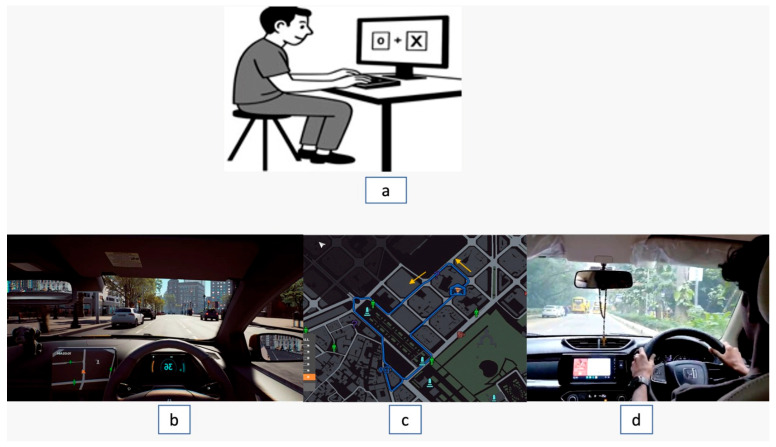
Experimental setups used across the three training modalities. (**a**) Illustration of a participant performing a computer-based cognitive task using the PsyToolkit platform to assess visuomotor coordination and attentional cueing. (**b**) A driving view from the game-based simulator used during training sessions. (**c**) Top-view map of the predefined simulator route followed by participants during the game-based training. (**d**) Real-world driving setup inside the vehicle during supervised on-road training along a fixed urban route.

The visual stimuli were presented on a calibrated 32-inch monitor with a high-resolution display (1920 × 1080), which was connected to a computer optimized for precise stimulus projection. Subjects were seated comfortably at an 80 cm distance from the monitor. Figure 1a: a photo of the experimental setup for PsyToolkit training.

Fitts’ law and cue-utilization tasks were selected based on pilot study results, which suggested that these measures were the most responsive to training-related changes.

#### 2.2.2. Valid and Invalid Cue Measurement Using the Posner-Cueing Task

This measures attentional reorientation using valid and invalid trials Figure 1a. Each trial began with two boxes flanking a central fixation point. In valid trials, an “×” cue appeared for 200 ms, followed by a 500 ms delay; in uncued trials, a 700 ms interval was used. A green-circle target then appeared at the cued or opposite location, and participants responded via the right-shift key. Reaction Time (RT) was recorded.

Fitts’ Law was designed to assess hand–eye coordination based on the classic Fitts’ Law paradigm, with outcomes including a measure combining speed and accuracy. Participants had to alternatively tap using keys or a mouse on two onscreen targets, which appeared at different places on the screen.

To capture overall visuomotor-attentional performance, a composite PsyToolkit score was computed by summing reaction times across the three cognitive-motor tasks. An improvement score was then calculated as the difference between post- and pre-training composite scores.

#### 2.2.3. Game-Based Simulator Training

Game-based simulator training was conducted on a 32-inch monitor with a high-resolution display (1920 × 1080) and a Nitho Drive PRO V16 multi-format racing wheel system. The racing wheel system consists of a 10-inch (255 mm) steering wheel with 270° rotation and an integrated rumbling motor for driving feedback. Steering sensitivity was adjustable across three levels. Gear control was provided through dual paddle shifters and a sequential gear shifter on the right side, while separate floor-mounted accelerator and brake pedals supported a realistic seated posture.

The driving scenario was implemented using *Taxi Life: A City Driving Simulator*. Participants completed a predetermined route between two fixed points that included traffic signals, lane changes, pedestrian crossings, parking tasks, and speed regulation Figure 1b. Photo of the driving simulator’s Figure 1c top view of the course; the arrow indicates the direction and starting location. Each session required approximately 12–15 min to complete.

Driving performance was automatically recorded by the simulator software, and the error metrics such as collisions, lane deviations, incorrect turns, speed violations, and signalling errors were calculated. The simulator route and traffic conditions were identical across sessions to ensure comparability across participants.

#### 2.2.4. Real-World Driving Training

Participants assigned to the real-world training group completed supervised driving sessions in a Honda Amaze 1.5 CVT automatic vehicle along a fixed urban route. The route covered approximately 5.5–6 km and included signalized intersections, pedestrian crossings, and a moderate level of traffic density.

Each session lasted approximately 15–17 min and was conducted under instructor supervision. Verbal feedback was provided after each session on hazard detection, mirror use, traffic compliance, and driving behaviour Figure 1d.

To ensure comparability across groups, all training sessions were conducted once daily for 10 consecutive days. Session durations were designed to be roughly equivalent across training modalities while maintaining ecological validity for each training condition.

#### 2.2.5. Eye-Tracking and Gaze Analysis

Eye movements were recorded using the Gaze Recorder platform (EyeTech Digital Systems, Mesa, AZ, USA), a webcam-based remote eye-tracking system operating at a nominal sampling rate of ~30 Hz with an estimated spatial accuracy of approximately 0.5–1.0° of visual angle under optimal calibration.

Before each recording session, a calibration procedure was conducted for each participant to ensure accurate gaze estimation. The quality of calibration was verified using the platform’s built-in validation procedure, and recalibration was conducted when necessary.

Participants viewed a standardized driving video depicting real-world traffic scenarios (e.g., vehicles, pedestrians, road signs, and environmental distractors) during both pre- and post-training assessments, and eye-tracking was recorded for the full duration of the stimulus presentation in each session.

Areas of Interest (AOIs) were predefined based on driving-relevant visual regions and included the dashboard, side mirrors, rear-view mirror, traffic signs and signals, pedestrians, and roadway ahead. Additionally, the gaze of drivers across various sectors like centre, left, right, superior-left, superior-right, inferior-left, inferior-right, up, and down regions was measured. AOIs were implemented as static divisions overlaid on the video to ensure consistency across participants and sessions, although this approach may not fully capture the dynamic nature of real-world driving environments.

Dwell time percentage was calculated as the proportion of total fixation duration falling within each AOI compared to the total fixation time across the stimulus presentation. Fixations were identified using the software’s default algorithm with a minimum duration threshold (~100 ms), and segments affected by blinks or excessive noise were excluded. Overall, data loss was minimal and did not differ much between pre- and post-training sessions, indicating comparable data quality across conditions.

To examine temporal changes in gaze allocation, the stimulus presentation was divided into consecutive 5 s intervals (0–5, 5–10, 10–15, …, 55–60 s). For each interval, dwell time within each AOI was calculated and averaged across participants for both pre- and post-training sessions. This analysis of dwell time for each AOI was calculated to assess how gaze behaviour changed over time during the video.

The eye-tracking system used in this study operated at a sampling rate of approximately 30 Hz and was appropriate for the objectives of the analysis. The study primarily focused on area of interest (AOI) measures, such as where participants looked and how long they fixated on different regions, rather than on very rapid eye movements like micro-saccades. In applied driving research, webcam-based eye trackers at this sampling rate have been shown to provide sufficiently reliable estimates of gaze allocation. While higher-frequency systems are better suited for detailed saccadic analysis.

### 2.3. Procedures

Following approval from the institutional ethics committee and acquisition of written informed consent, all participants underwent a preliminary ocular examination and provided demographic information, including age, sex, driving experience, and refractive status. Baseline measurements were obtained for all three groups across visual–cognitive performance, simulated driving errors, and gaze-based scanning behaviour, as summarized in Table 1.

### 2.4. Baseline Assessments

Baseline testing comprised four components:

1. Visual–Cognitive Performance (PsyToolkit): Participants completed computerized tasks assessing hand–eye coordination and attentional cue utilization, including valid and invalid cue paradigms;
2. Driving Error Measurements (Game-Based Simulator): Performance was quantified using predefined error categories: wrong turns, collisions, reversing-related accidents, steering corrections, speed violations, lane deviations, failure to stop after crossing signals, and indicator-use errors;
3. Gaze-Based scanning by Areas of Interest (AOIs): Eye-movement data were collected for the following AOIs: dashboard, side mirrors, rear-view mirror, traffic signals and signs, pedestrians, roadway ahead, and roadside billboards;
4. Gaze-Based scanning by Spatial Sectors: Fixations were further classified into centre, left, right, up, down, superior left and right, and inferior left and right sectors.

### 2.5. Group Allocation and Training Protocol

Participants were assigned to one of three independent training groups, each focusing on a distinct aspect of driving-related skill acquisition:

- Group 1—Visual and Cognitive Training (PsyToolkit): Participants in Group 1 completed computerized attentional training using PsyToolkit software. Training emphasized the utilization of valid and invalid spatial cues and hand-eye coordination. Sessions were conducted once daily for 10 consecutive days. Each session was preceded by task familiarization and followed by brief feedback regarding the accuracy of response and reaction times.
- Group 2—Game-based simulator training: Group 2 underwent training using a game-based driving simulator (Nitho Drive Pro steering system, Version 1). Participants completed standardized driving scenarios across 10 consecutive daily sessions. During each session, driving performance was monitored and quantified using the predefined error metrics listed above. At the conclusion of every training session, structured feedback was provided regarding the nature and frequency of errors committed, with emphasis on lane discipline, speed control, signalling behaviour, and collision.
- Group 3—Real-World Driving Training: Participants in Group 3 received on-road training in a Honda CVT automatic transmission vehicle along a predetermined route selected to include multiple critical AOIs, such as traffic signals, pedestrian crossings, intersections, road signs, and merging zones. Training was conducted for 15 min once daily for 10 consecutive days under instructor supervision. Feedback was delivered after each drive, focusing on hazard detection, mirror use, compliance with traffic signals, pedestrian awareness, and intersection negotiation.

Prior to commencement of formal training, all participants were given sufficient familiarization time with their assigned training modality—computer-based tasks, simulator controls, or vehicle handling—to minimize learning effects related to equipment use. Across all three groups, verbal feedback was provided following every session to reinforce correct behaviours and to highlight specific errors or inefficiencies observed during training.

Upon completion of the 10-day training period, all baseline measures were repeated for every participant. Visual–cognitive performance was reassessed using PsyToolkit tasks, driving errors were re-evaluated in the simulator, and gaze distribution across AOIs and spatial sectors was recorded using eye-tracking methods identical to those employed at baseline. Pre- and post-training outcomes were subsequently tabulated and subjected to statistical analysis.

To minimize potential confounding effects, training conditions were standardized across all groups in terms of session duration, frequency (10 consecutive days), and task structure. Simulator scenarios and real-world driving routes were fixed across participants, and all sessions were supervised using a consistent protocol.

The primary outcomes of the study were defined as composite PsyToolkit improvement scores and key AOI dwell-time measures, while sector-based and scan-order metrics were treated as secondary exploratory outcomes. This approach was adopted to reduce the number of statistical comparisons and limit type I error to focus interpretation on theoretically relevant endpoints. To further control for Type I error arising from multiple comparisons, analyses were structured around these predefined outcome domains, and Bonferroni-adjusted post hoc tests were applied where appropriate.

Parametric tests were used for normally distributed data, while non-parametric tests were applied when normality assumptions were violated.

## 3. Results

Results are presented with emphasis on predefined primary outcomes, followed by secondary exploratory analyses. Normally distributed variables are reported as mean ± SD, whereas non-normal variables are reported as median (IQR). Primary outcomes included composite PsyToolkit scores and AOI dwell-time measures, whereas scan-order, sector-based, and time-interval analyses were treated as secondary exploratory outcomes. Effect sizes are reported where appropriate. For non-parametric analyses, effect magnitudes were interpreted based on Z values, with larger absolute Z values indicating stronger effects.

### 3.1. Baseline Characteristics

The mean age of participants was 21.13 ± 2.03 years, and the mean driving experience was 0.74 ± 0.17 years. Median refractive error was 0.00 D (IQR = 0.63), and median weekly driving hours were 3 h (IQR = 2.25). There were no significant differences between groups in age, F(2,27) = 2.28, *p* = 0.122, or driving experience, F(2,27) = 0.01, *p* = 0.987. Kruskal–Wallis tests indicated no group differences for refractive error (*p* = 0.526) or hours of driving per week (*p* = 0.993). Gender distribution did not differ significantly between groups, χ^2^(2) = 4.29, *p* = 0.117.

### 3.2. PsyToolkit Training

**Figure 2:**
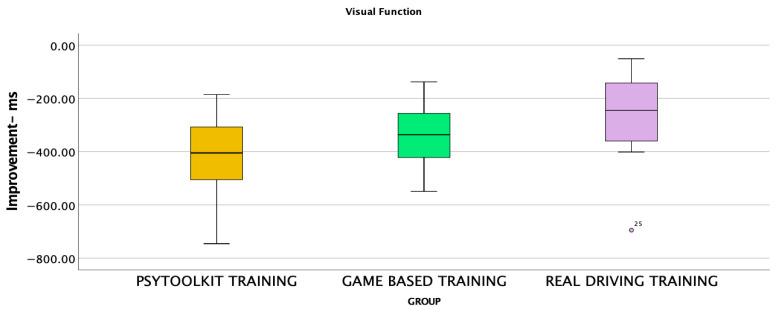
Boxplot showing composite PsyToolkit improvement scores (post−pre) across the three training groups. Negative values indicate greater reductions in reaction time (i.e., greater improvement). Boxes represent the interquartile range (IQR), horizontal lines indicate medians, whiskers denote minimum and maximum values. Individual data points are overlaid; the labelled point (“25”) represents a participant ID and indicates an outlier within the real-driving group.

Pre-training performance did not differ between groups across PsyToolkit measures, indicating baseline equivalence (Table 2). Mixed repeated-measures ANOVA revealed a significant main effect of Time, F(1,27) = 123.44, *p* < 0.001, η^2^p = 0.82, demonstrating overall improvement following training, as well as a significant Time × Group interaction, F(2,27) = 4.14, *p* = 0.027, η^2^p = 0.23, indicating that improvement differed across training conditions, whereas the main effect of Group was not significant, F(2,27) = 0.30, *p* = 0.740. Post-training comparisons showed no group differences for valid- or invalid-cue reaction times. However, significant group effects were observed for Fitts’ Law performance and the composite improvement score. Tukey post hoc tests indicated that the PsyToolkit-only group performed significantly faster than the real-driving group for Fitts’ Law RT (mean difference = −97.06 ms, *p* = 0.028) and for the composite PsyToolkit score (mean difference = −175.05, *p* = 0.024), while all other pairwise comparisons were non-significant (*p* > 0.05). The distribution of composite PsyToolkit improvement scores across the three training groups is illustrated in Figure 2, highlighting greater improvements in the cognitive training group. The use of a composite PsyToolkit score provided an important measure of visuomotor and attentional performance, reducing reliance on multiple individual comparisons.

### 3.3. Game-Based Training

**Figure 3:**
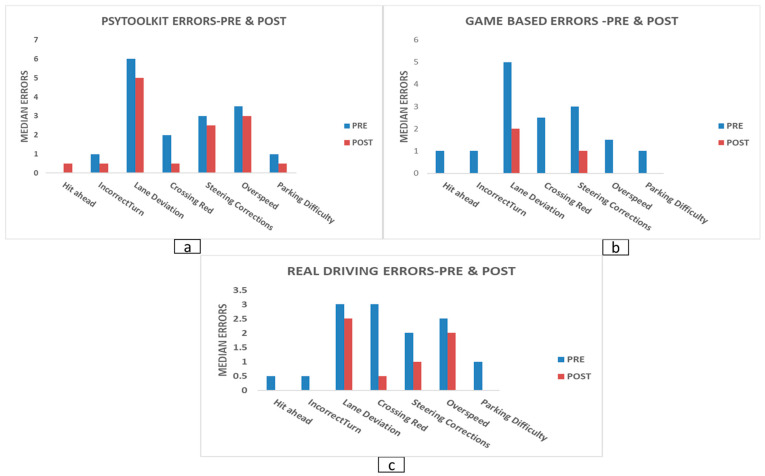
Comparison of median simulator driving errors before (Pre) and after (Post) training across the three training modalities. (**a**) PsyToolkit-based cognitive training, (**b**) game-based simulator training, and (**c**) real-world driving training. Blue bars represent pre-training medians, and orange bars represent post-training medians. Variables with zero medians in both pre and post were excluded to improve interpretability.

Because normality assumptions were violated, non-parametric analyses were used. Kruskal–Wallis tests on pre–post change scores showed significant group effects for several driving variables. These included hit-vehicle-ahead during turning (*p* = 0.003), incorrect turns on one-way roads (*p* = 0.005), wrong turns (*p* = 0.043), lane deviation (*p* = 0.002), failure to indicate (*p* = 0.043), crossing after a red signal (*p* = 0.018), and stopping before the signal (*p* = 0.038). No significant group effects were observed for missed pedestrians, hard braking, steering corrections, overspeeding, accident counts, or parking difficulty (all *p* > 0.005). Mann–Whitney post hoc tests indicated that game-based simulator training produced greater reductions than PsyToolkit training for hit-vehicle-ahead (U = 12.0, Z = −2.99, *p* = 0.003), incorrect turns (U = 16.5, Z = −2.64, *p* = 0.008), wrong turns (U = 26.0, Z = −1.95, *p* = 0.050), lane deviation (U = 12.5, Z = −2.89, *p* = 0.004), failure to indicate (U = 18.0, Z = −2.56, *p* = 0.010), crossing after a red signal (U = 19.5, Z = −2.35, *p* = 0.018), and stopping before the signal (U = 20.5, Z = −2.29, *p* = 0.022). Game-based simulator training also outperformed real-world driving for incorrect turns (U = 15.0, Z = −2.85, *p* = 0.004), wrong turns (U = 21.5, Z = −2.37, *p* = 0.018), and lane deviation (U = 7.5, Z = −3.27, *p* = 0.001). Overall, the game-based intervention was associated with consistent improvements across safety-critical driving behaviours. Changes in simulator-based driving errors before and after training across groups are presented in Figure 3, demonstrating the largest reductions in errors in the game-based training group.

### 3.4. Dwell Percentage AOI Results

Preliminary assessment of distributional assumptions using Shapiro–Wilk tests indicated that several gaze-dwell variables deviated from normality across training groups. Given the small sample size (*n* = 10 per group) and these violations, all subsequent analyses were conducted using non-parametric procedures.

Training-related change scores were calculated for each AOI as post-training minus pre-training dwell time (Δ). Between-group differences in these change scores were examined using Kruskal–Wallis tests, followed by Bonferroni-adjusted pairwise Mann–Whitney U comparisons (α = 0.017). In addition, overall pre–post changes across all participants (N = 30) were evaluated using Wilcoxon signed-rank tests.

Independent-samples Kruskal–Wallis tests conducted on baseline dwell-time values confirmed that the three training groups did not differ significantly prior to intervention for any AOI (all *p* > 0.05), indicating successful group matching at pre-test.

Post-training Kruskal–Wallis analysis revealed significant group differences for dashboard (*p* = 0.014), side-mirror (*p* = 0.043), car (*p* < 0.001), road-ahead (*p* = 0.010), rear-view (*p* = 0.002), and pedestrian AOIs (*p* = 0.001). Sign and billboard regions did not differ significantly after training (*p* > 0.05).

**Figure 4:**
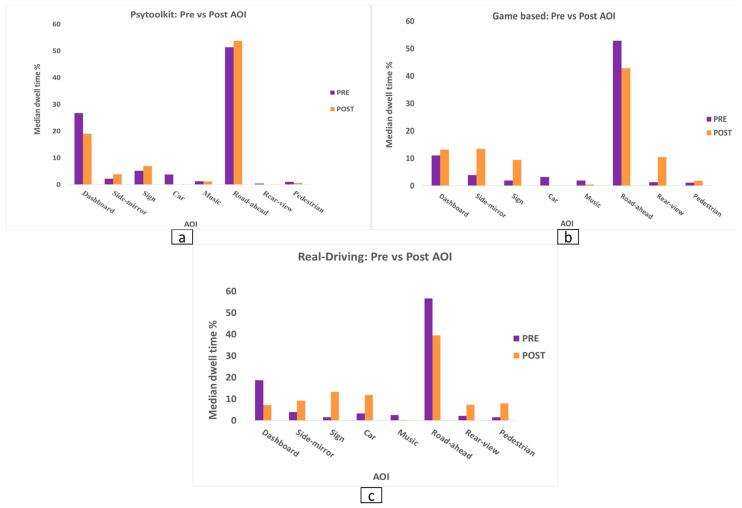
Pooled median dwell time percentages across all participants before and after training for each AOI. (**a**) PsyToolkit-based cognitive training, (**b**) game-based simulator training, and (**c**) real-world driving training. Purple bars represent pre-training values and orange bars represent post-training values.

Across all training groups, Wilcoxon signed-rank tests demonstrated significant reductions in dashboard-related gaze (Z = −2.60, *p* = 0.009) and music-related gaze (Z = −2.38, *p* = 0.018). In contrast, significant increases in dwell time were observed for several safety-relevant AOIs, including side-mirror (Z = −4.29, *p* < 0.001), traffic-sign (Z = −3.92, *p* < 0.001), rear-view mirror (Z = −2.91, *p* = 0.004), and pedestrian regions (Z = −2.63, *p* = 0.009). No significant overall changes were found for car, road-ahead, or billboard AOIs (all *p* > 0.05). The pooled pre–post changes in dwell time across areas of interest are shown in Figure 4, illustrating reduced attention to in-vehicle regions and increased monitoring of safety-relevant AOIs.

Bonferroni-adjusted Mann–Whitney U tests indicated that PsyToolkit training produced significantly greater improvement in road-ahead dwell time than rrreal-driving training (U = 12.0, *p* = 0.004). Conversely, Real-driving training yielded significantly larger gains for rear-view mirror monitoring (U = 18.0, *p* = 0.015) and traffic-sign fixation (U = 12.0, *p* = 0.004). Although the game-based group demonstrated improvements across multiple AOIs, none of its pairwise comparisons remained significant after Bonferroni correction.

Overall, training produced beneficial changes in gaze behaviour across participants, characterized by reduced attention to in-vehicle distractions and increased monitoring of safety-critical regions. However, the extent of these improvements depended on training type: real-driving training was associated with greater improvements in pedestrian and sign monitoring and rear-view mirror use, whereas PsyToolkit training primarily enhanced forward-scene scanning.

### 3.5. Dwell AOI-First View

Median first-view ranks (ordinal data) across twelve 5 s intervals were calculated for each participant and AOIs, with lower values representing earlier fixation in the scan sequence. When pooled across all participants, Wilcoxon signed-rank tests revealed a statistically significant pre–post change only for the dashboard AOI (Z = −2.74, *p* = 0.006), reflecting delayed dashboard prioritization following training. No significant overall changes were observed for side mirrors, signs, car interior, music controls, road-ahead region, rear-view mirrors, or pedestrians (all ps > 0.17).

Group-wise analyses revealed differential effects of the three training paradigms. In the PsyToolkit group, dashboard first-view rank increased significantly from pre- to post-training (median = 2.0 [1.0–2.0] vs. 2.0 [2.0–2.5]; Z = −2.26, *p* = 0.024). The game-based simulator training group did not exhibit statistically reliable changes for any AOI (all ps > 0.19). In contrast, real-driving training produced significant changes in fixation order for mirror-related AOIs. First-view rank increased for both the rear-view mirror (median = 2.5 [1.0–3.5] vs. 4.0 [4.0–4.5]; Z = −2.21, *p* = 0.027) and side mirrors (median = 2.0 [2.0–2.5] vs. 3.0 [3.0–3.5]; Z = −1.97, *p* = 0.049), indicating later mirror fixation following training. Trend-level increases were also observed for the road-ahead region and music controls, suggesting broader shifts in visual scanning sequence after real-world driving exposure.

Between-group comparisons conducted on pre–post change scores using Kruskal–Wallis tests demonstrated a statistically significant effect of training group only for rear-view mirror prioritization (*p* = 0.024), indicating that the magnitude of training-related change differed across PsyToolkit, game-based, and real-driving groups. No significant between-group differences were detected for dashboard, side mirrors, signs, car interior, music, road-ahead region, or pedestrians (all *p* > 0.15). The detailed distribution of first-view (scan-order) ranks across AOIs for each training group is presented in Table 3. Overall, scan-order changes were limited, with only a small number of AOIs showing significant differences, suggesting that training effects on temporal gaze sequencing were modest compared to dwell-time adaptations.

### 3.6. Dwell Percentage Sector Analysis

Normality testing indicated departures from Gaussian distributions; therefore, non-parametric analyses were applied. Friedman’s analysis of AOI change scores demonstrated significant redistribution across spatial regions (*p* < 0.001), and Bonferroni-corrected post hoc comparisons showed that centre differed from superior-right (Z = −4.81, *p* < 0.001) and left (Z = −5.04, *p* < 0.001), inferior-right differed from superior-right (Z = 3.75, *p* = 0.006) and left (Z = 3.98, *p* = 0.002), and right differed from superior-right (Z = −3.35, *p* = 0.029) and left (Z = 3.58, *p* = 0.012), with all other pairwise contrasts remaining nonsignificant after correction. Between-group comparisons of dwell-time change scores using Kruskal–Wallis tests revealed no significant differences among the three training conditions for any AOI. Because no omnibus between-group effects were detected, follow-up Mann–Whitney tests (U statistics) were not performed. These results indicate that although gaze distribution shifted following training, the magnitude and spatial pattern of redistribution were comparable across the three interventions. This lack of between-group differences may reflect the limited sensitivity of the study design and sample size rather than the absence of true effects. Pre–post median dwell time values across spatial sectors for each group are provided in Table 4.

**Figure 5:**
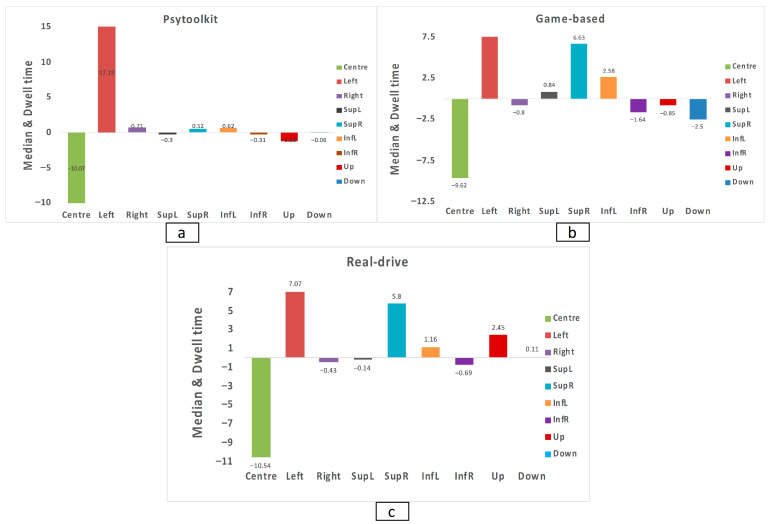
Median change (Δ = post–p post-pre) in dwell time across areas of interest (AOIs) for (**a**) PsyToolkit-based cognitive training, (**b**) game-based simulator training, and (**c**) real-world driving training. Positive values indicate increased post-training gaze allocation; negative values indicate decreases. The horizontal line marks zero change.

The median change in dwell time across spatial sectors for each training group is illustrated in Figure 5, showing the redistribution of gaze following training.

### 3.7. Time-Interval Analysis of AOI Dwell Across Training Modalities

**Figure 6:**
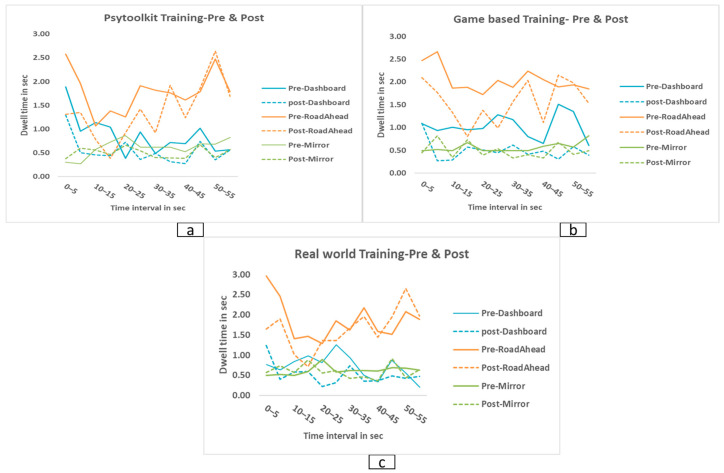
Pre- and post-training gaze dwell time across 12 time intervals (0–60 s) for different areas of interest (AOIs) in the (**a**) PsyToolkit (cognitive), (**b**) Game-based, and (**c**) real-world driving training groups. Solid lines represent pre-training values and dashed lines represent post-training values. Across training conditions, post-training gaze patterns show reduced attention to in-vehicle regions and increased monitoring of safety-relevant areas such as mirrors, pedestrians, and traffic signs.

Across the three training groups, gaze dwell time varied across the 0–60 s viewing sequence but consistently showed the greatest allocation toward the road-ahead region across most intervals. In the PsyToolkit (cognitive) training group, dashboard dwell time was higher during the early intervals (0–10 s) but decreased after training. Attention to the road-ahead region, on the other hand, stayed stable and strong during the mid and later intervals (25–55 s). In the game-based simulator training group, post-training traces indicated reduced dashboard dwell time and modest increases in gaze toward mirrors and pedestrians, particularly during the mid-sequence intervals (20–40 s). In the real-world driving training group, post-training traces showed increased dwell time toward mirrors and traffic-relevant peripheral regions during the later intervals (35–55 s), while the road-ahead region continued to receive the highest gaze allocation throughout the sequence. Temporal changes in gaze allocation across the 0–60 s intervals for each training modality are depicted in Figure 6, demonstrating dynamic shifts in attention over time. These time-interval analyses were descriptive in nature and were not subjected to formal statistical modelling; therefore, the observed temporal patterns should be interpreted cautiously.

Figures were refined to enhance clarity by emphasizing key outcome variables and reducing less informative elements where appropriate.

## 4. Discussion

This study examined how three training modalities influenced gaze behaviour and driving-related performance in novice drivers using a pre–post design. Across all training conditions, participants showed reduced attention to in-vehicle distractions and increased monitoring of safety-relevant regions such as mirrors, pedestrians, and traffic signs. Changes in gaze behaviour following training are commonly interpreted within frameworks of scan-path plasticity, in which repeated task exposure reorganizes both the spatial distribution and temporal sequencing of fixations. Early work by Alfred Yarbus demonstrated that eye movements are shaped by task demands and are increasingly understood as flexible, task-dependent strategies rather than fixed routines [20]. In driving, expertise has been associated with broader visual scanning, earlier monitoring of potential hazards, and reduced attention to in-vehicle distractions [21,22].

Training-induced changes in eye movements have been documented in hazard-perception programmes [16], simulator-based instruction, and perceptual–cognitive interventions [23]. The present findings suggest that different training modalities were associated with distinct patterns of gaze allocation rather than a single expert-like scanning pattern.

This aligns with prior research demonstrating that eye-tracking metrics reflect driver situation awareness and adapt to task demands, environmental complexity, and training exposure [24,25,26]. This is consistent with previous work showing that gaze behaviour adapts to task demands and training exposure. However, these changes should be interpreted as modifications in gaze allocation rather than definitive indicators of improved driving performance or safety.

### 4.1. Cognitive Training and Visuomotor Efficiency

The use of PsyToolkit training for Fitts’ Law performance is consistent with laboratory studies showing that repeated cueing and pointing tasks refine attentional orienting and motor planning networks [27]. Researchers have also reported the transfer of this cognitive training to visually guided actions in driving. Visual attention training can speed responses and improve manual performance even when overall scanning patterns change only modestly [28]. This suggests that cognitive drills often enhance the efficiency of visual–motor performance rather than fundamentally reorganizing spatial search strategies [29]. PsyToolkit training was associated with greater improvements in road-ahead dwell time. However, it did not strongly enhance mirror or peripheral monitoring. This suggests optimization of central hazard processing rather than distributed search.

### 4.2. Simulator Training and Driving Behaviour

Game-based simulator training was associated with notable reductions in simulator driving errors, particularly for lane keeping and signalling behaviours [30,31]. This is consistent with hazard-perception and self-confidence research by GD Park, who showed that simulator-trained novice drivers had better hazard perception and higher driver self-confidence than drivers with no training [32]. Simulator-based training increases anticipatory glances toward latent threat locations and broader visual exploration following expertise training [29,33]. The game-based group showed widespread improvements without a dominant change in any single AOI. This suggests an overall shift in visual attention rather than a localized adaptation. Results show short-term real-world exposure may alter how visual attention is distributed across the scene without immediately producing expert-like scanning behaviour.

### 4.3. Real-World Driving and Gaze Adaptations

Real-world training was associated with increased dwell time toward mirrors and traffic-relevant peripheral regions. Scan-order analyses, however, showed that mirror fixation happened later, not earlier, after training. Previous naturalistic driving studies show that such behaviours develop gradually with on-road experience, with experienced drivers sampling mirrors and roadside information more frequently than novices [34].

### 4.4. AOI Redistribution and Modality-Specific Visual Tuning

Across training modalities, participants reduced gaze on dashboard and music controls while increasing attention to pedestrians, signs, and mirrors—changes long associated with expert drivers [35,36]. Crundall and Underwood (1998) similarly reported that novices mostly fixate near the vehicle front, whereas experienced drivers distribute gaze toward potential hazard zones [35].

### 4.5. The Modality-Specific AOI Effects

PsyToolkit training improved forward monitoring, real-world training enhanced rearward and lateral gaze surveillance, and simulator training produced moderate but widespread improvements, although these did not remain statistically superior after correction for multiple comparisons [37,38]. It is important to distinguish between findings that were strongly supported by statistical evidence and those that should be interpreted as exploratory. Robust effects were observed for key outcomes such as reductions in simulator driving errors and changes in AOI dwell time (e.g., increased monitoring of mirrors and pedestrians). In contrast, changes in scan-order (first-fixation measures) and sector-based gaze distribution were less consistent and should be considered exploratory trends that require further validation in larger samples.

### 4.6. Scan-Order Versus Dwell-Time Adaptations

Fewer changes were observed in scan-order measures than in dwell-time indices. This suggests that training primarily influenced how long drivers looked at different areas, while overall scanning order remained largely unchanged. This pattern aligns with prior eye-movement studies that demonstrate early learning typically alters fixation duration prior to more extensive modifications in scan-path structure [36]. The limited number of significant findings in scan-order measures suggests that training effects on temporal gaze sequencing were modest, and underlying mechanisms remain speculative [21].

Across all participants, training led drivers to delay looking at the dashboard, indicating that they maintained attention on the roadway for longer before shifting to in-vehicle displays. Distraction-reduction studies in Transportation Research Part F have reported similar effects. In addition, participants in the real-world training group showed later mirror fixation in the scan sequence, suggesting that drivers prioritized forward roadway monitoring before sampling mirror information, consistent with a study on drivers with longer on-road glances and fewer glance shifts that had fewer crashes than near-crashes [39].

Post-training changes showed that participants looked more often toward the sides and upper areas of the visual scene, a pattern that resembles the wider visual scanning reported in experienced drivers [36]. Because this shift was observed in all three training groups, it likely reflects a general effect of structured practice rather than a consequence of any single intervention. At the same time, the three training methods influenced visual attention to different areas of interest, suggesting that gaze allocation varied depending on the training modality.

### 4.7. Contribution of the Present Study

The present study contributes to the existing literature in several important ways. (1) a direct comparison of cognitive, simulator, and real-world training within a single framework; (2) integration of multiple gaze metrics (AOI dwell time, sector analysis, and first-view/scan-order); and (3) identification of modality-specific gaze adaptations. The study also shows that training has a greater effect on dwell-time rather than scan-order, highlighting early changes in fixation behaviour. These results extend current knowledge on how different training approaches shape visual attention in novice drivers. Recent longitudinal and meta-analytic studies further support the role of training in shaping hazard perception and gaze behaviour over time, highlighting the importance of sustained and multimodal interventions [40].

### 4.8. Limitations

The relatively small sample size (*n* = 10 per group) represents a limitation and may reduce statistical power. However, several significant findings showed large effect magnitudes (e.g., Z ≈ −2.9), suggesting adequate sensitivity for detecting robust effects. The study may nevertheless have been underpowered to detect small-to-moderate effects, particularly in between-group comparisons where eye-tracking measures show high inter-individual variability. As a result, the present sample size may not have been sufficient to detect smaller differences between training groups. Caution is warranted when extrapolating these findings to broader driver training recommendations, particularly given the modest sample size and short training duration.

Additionally, eye movements were recorded using a webcam-based eye-tracking system operating at approximately 30 Hz. While this system provides sufficient accuracy for area of interest analysis, it may be less sensitive than high-frequency laboratory eye trackers for detecting rapid saccadic events and precise fixation timing, which may affect scan-order (first-fixation) analyses. Therefore, scan-order findings should be interpreted with caution. The absence of a follow-up assessment limits conclusions regarding long-term retention and real-world transfer of training effects.

Furthermore, the use of static areas of interest (AOIs) may not fully capture the dynamic nature of real-world driving environments, where relevant objects continuously change position. This may limit the ecological validity of gaze allocation measures. Finally, the training duration was limited to ten sessions, and longer interventions may produce stronger or more stable gaze adaptations. Future studies should incorporate longer training periods and higher-resolution eye-tracking systems to measure rapid saccadic eye movements during the training of drivers. Despite these precautions, the possibility of residual Type I error due to multiple comparisons cannot be fully excluded.

### 4.9. Practical Recommendation and Future Work

From an applied standpoint, the results argue against a single-method approach to novice driver education and training. Instead, this suggests that a multimodal approach to driver training in which cognitive exercises, game simulation, and supervised on-road experience target various components of visual behaviour and performance may be beneficial, although further evidence is required before firm recommendations can be made. Eye-tracking metrics—particularly AOI dwell patterns and scan-order indices—are well suited for guiding such individualized training programmes by identifying specific visual areas that require improvement.

Future work should look at whether combining these training modalities yields further benefits, and whether the observed gaze adaptations persist over time and help in real-world driving. To better understand these results, larger samples and longer training times will be necessary.

## 5. Conclusions

This study suggests that training altered how novice drivers looked at the road and how they drove; however, different training methods led to different kinds of improvements. Across all groups, training reduced attention to in-vehicle distractions and promoted broader visual exploration toward safety-critical regions, indicating overall improvements after training. At the same time, each training method was associated with changes in gaze behaviour in different ways: cognitive training primarily enhanced visuomotor efficiency and forward-scene monitoring; simulator-based training yielded the largest reductions in tactical driving errors; and real-world driving strengthened mirror use, sign monitoring, and early peripheral scanning.

Eye-movement measures were sensitive to differences between training methods. AOI-based analyses showed that training altered gaze allocation toward specific driving-relevant areas (e.g., mirrors, pedestrians, and signs). In contrast, sector-based analyses indicated a broader spatial distribution of gaze, suggesting that visual search became more widely distributed across the scene following training. Training reveals that driving is multi-dimensional, involving where they looked, how widely they scanned, and when they looked—not just one single skill.

## Figures and Tables

**Table 1:** Baseline outcome measures and training-specific parameters assessed across the three experimental groups.

Visual Function	Game-Based Training Parameters	Gaze-Based Scanning-AOI	Gaze-Based Scanning-Sector
Hand–eye co-ordination Valid and Invalid cue	Wrong turns Accidents Accidents while reversing Steering corrections Speed violations Lane deviations Stopping after crossing signal Indicator errors	Dashboard Sidemirrors Rear mirrors Signal & Signs Pedestrians Gaze ahead Billboards	Centre Right and Left Up and Down Superior right & left Inferior right & left

**Table 2:** Summary of PsyToolkit results across training groups.

Parameter	Time	PsyToolkit Training	Game-Based Simulator Training	Real-Driving Training	*p*-Value (Group)
Valid-cue RT	Pre	486.27 ± 35.82	473.88 ± 46.47	469.28 ± 43.12	0.651
	Post	363.75 ± 37.38	388.73 ± 35.07	403.60 ± 39.49	0.072
Invalid-cue RT	Pre	596.87 ± 52.10	553.49 ± 65.68	568.23 ± 59.40	0.268
	Post	460.09 ± 42.82	471.02 ± 46.86	498.23 ± 53.67	0.206
Fitts’ Law RT	Pre	813.09 ± 59.64	864.21 ± 85.00	881.35 ± 105.39	0.197
	Post	640.68 ± 79.72	703.01 ± 80.23	737.74 ± 77.26	0.033 *
Composite PsyToolkit improvement score	Post-Pre	−431.7 ± 165.1	−328.8 ± 127.2	−279.3± 187	0.031 *

Note: RT = reaction time (milliseconds, ms). Significant *p*-values marked with *. *p*-values reflect omnibus ANOVA tests; pairwise Tukey post-hoc *p*-values are reported in the text.

**Table 3:** Median (IQR) first-view rank across training groups and sessions. (Lower values indicate earlier first fixation in the scan sequence).

AOI	PsyToolkit Pre	PsyToolkit Post	Game Pre	Game Post	Real-Driving Pre	Real-Driving Post	*p*-Value *
Dashboard	2.0 (1.0–2.0)	2.0 (2.0–2.5)	2.0 (2.0–2.0)	2.0 (2.0–2.5)	2.0 (2.0–3.0)	3.0 (2.5–3.0)	0.00 †
Side Mirror	2.0 (0–2.5)	2.25 (2.0–2.5)	2.75 (2.0–3.0)	2.5 (2.0–3.0)	2.0 (2.0–2.5)	3.0 (3.0–3.5)	0.04 ‡
Sign	2.7 (1.5–3)	1.75 (1.0–2.0)	2.0 (2.0–3.0)	2.75 (2.0–3.0)	3.0 (1.5–3.25)	3.0 (2.75–3.75)	0.46
Rear-View	1.0 (0–3.0)	0.50 (0–2.0)	2.0 (0–4.0)	2.25 (2.0–3.0)	2.5 (1.0–3.5)	4.0 (4.0–4.5)	0.02 §
Road Ahead	1.0 (1.0–2.0)	1.0 (1.0–1.0)	1.0 (1.0–2.0)	1.5 (1.0–2.0)	1.5 (1.0–1.75)	2.0 (1.75–2.0)	0.23
Music	3.0 (2.0–3.0)	2.75 (0–3.0)	3.0 (2.5–4.0)	2.0 (0–3.0)	3.0 (3.0–3.75)	4.5 (3.75–4.75)	0.15
Pedestrian	2.0 (0–2.5)	1.5 (0–2.0)	2.0 (0–3.0)	2.0 (2.0–4.0)	3.0 (2.0–3.0)	2.0 (1.0–3.75)	0.59

* *p*-values derived from Wilcoxon signed-rank tests (within groups) and Kruskal–Wallis tests on change scores between groups. † Significant pooled pre–post change across all participants. ‡ Significant pre–post change in the real-driving group. § Significant between-group difference in training-related change.

**Table 4:** Pre–post median dwell time by AOI and training group.

AOI	PsyToolkit Pre	PsyToolkit Post	Game Pre	Game Post	Real Pre	Real Post	*p*-Value
Centre	66.7 [23.4]	55.6 [23.6]	53.1 [24.8]	43.6 [18.2]	62.8 [23.1]	52.4 [10.4]	0.97
Left	4.1 [8.6]	21.3 [23.2]	10.3 [12.7]	17.9 [17.2]	9.3 [11.0]	19.6 [15.3]	0.77
Right	4.3 [5.1]	4.2 [2.5]	5.8 [6.5]	5.8 [3.0]	5.8 [5.0]	4.9 [4.5]	0.73
SupL	0.4 [1.6]	0.0 [2.0]	0.4 [2.2]	1.0 [6.5]	0.7 [1.9]	1.2 [4.4]	0.86
SupR	0.9 [2.1]	2.4 [6.1]	1.4 [6.4]	9.4 [5.4]	3.1 [5.4]	7.9 [6.0]	0.12
InfL	1.5 [3.2]	3.1 [4.0]	1.6 [2.5]	3.0 [4.7]	0.9 [5.6]	4.2 [4.0]	0.96
InfR	0.7 [1.8]	0.1 [1.3]	1.7 [4.0]	0.0 [0.5]	1.1 [0.7]	0.1 [0.4]	0.29
Up	2.7 [6.3]	1.2 [6.1]	5.2 [6.3]	6.2 [9.2]	1.2 [5.0]	5.6 [9.1]	0.61
Down	7.8 [17.8]	5.7 [9.9]	4.1 [13.8]	3.2 [4.6]	2.8 [3.4]	2.6 [5.7]	0.60

Note: Values are median [IQR]; *p* values from Kruskal–Wallis tests on delta scores. Non-parametric analyses were used due to non-normal distributions. Kruskal–Wallis tests indicated no significant between-group differences in dwell-time change scores across AOIs (all *p* > 0.05).

## Data Availability

The original contributions presented in this study are included in the article. Further inquiries can be directed to the corresponding author.

## Abbreviations

The following abbreviations are used in this manuscript:

AOI	Area of Interest
RT	Reaction Time
IQR	Interquartile Range
SD	Standard Deviation
